# Interkulturelle Kompetenzen im Gesundheitswesen durch Fort- und Weiterbildungen fördern

**DOI:** 10.1007/s00103-023-03768-3

**Published:** 2023-09-18

**Authors:** Desislava Dimitrova, Jalid Sehouli

**Affiliations:** Klinik für Gynäkologie mit Zentrum für onkologische Chirurgie, Europäisches Kompetenzzentrum für Eierstockkrebs (EKZE), Charité Comprehensive Cancer Center (CCCC), Charité Universitätsmedizin Berlin, Augustenburger Platz 1, 13353 Berlin, Deutschland

**Keywords:** IPIKA, Diversität, Gesundheitssystem, Interkulturelle Kommunikation, Kultursensible Versorgung, IPIKA, Diversity, Healthcare system, Intercultural communication, Culturally sensitive care

## Abstract

Die Akzeptanz der kulturellen Vielfalt und das Anerkennen und Wertschätzen der Facetten des „Andersseins“ wie Geschlecht, Alter, Berufsgruppe, Krankheitsgruppe, Religion, soziokultureller Hintergrund sowie Migrationsgeschichten sind Grundvoraussetzungen für eine adäquate Kommunikation und Interaktion im Gesundheitswesen. Der Begriff interkulturelle Kompetenz ist multidimensional und umfasst eine Reihe von Eigenschaften und Fähigkeiten, die sowohl emotionale und kognitive Elemente beinhalten als auch die Verhaltensebene berühren. In dem vorliegenden Artikel diskutieren wir die Bedeutung der interkulturellen Kompetenz für das Gesundheitswesen und welche Aspekte der interkulturellen Kommunikation für eine kultursensible medizinische Versorgung besonders relevant sind. Wir berichten außerdem über die Implementierung eines interprofessionellen Fort- und Weiterbildungskonzeptes für die klinische Praxis (IPIKA – „Interprofessionelles und Interkulturelles Arbeiten in Medizin, Pflege und Sozialdienst“).

Basis der spezifischen Ansätze zur Verbesserung der interkulturellen Kompetenz sollte die systematische Aus- und Weiterbildung grundsätzlicher kommunikativer Fähigkeiten sein. Dies betrifft nahezu alle Ausbildungscurricula für die medizinischen Professionen. Es ist wichtig zu betonen, dass interkulturelle Kompetenz ein Kern- und Querschnittsthema für alle Beteiligten im Gesundheitssystem darstellt.

## Abstract (en)

Acceptance of cultural diversity and the recognition and appreciation of facets of “otherness” such as gender, age, occupational or disease-related group, religion, sociocultural background, and migration history are basic requirements for adequate communication and interaction in healthcare. The term intercultural competence is multidimensional and includes a range of qualities and skills with emotional and cognitive elements with influence on the behavioral level. In the present paper, we discuss the importance of intercultural competence for the healthcare system and which aspects of the intercultural communication are particularly relevant for culturally sensitive medical care. Additionally, we report on the implementation of an interprofessional program and continuing education concept for clinical practice: Interprofessional and Intercultural Work in Medicine, Nursing and Social Services (IPIKA).

The basis of the specific approaches to improving intercultural competence should be the systematic education and training of basic communication skills. This concerns almost all curricula for the medical professions. It is important to emphasize that intercultural competence is a core and cross-sectional topic for all healthcare providers.

## Einleitung

Die globale Entwicklung führt zu einer ethnischen und kulturellen Vielfalt unserer Gesellschaft. Die kulturelle Diversität und der Pluralismus spiegeln sich auch im Gesundheitssystem wider und stellen neue Herausforderungen und Chancen sowohl für die Patient*innen als auch für das medizinische Personal dar.

Verschiedene nationale Studien berichten von Ungleichheiten in der medizinischen Versorgung von Patient*innen mit Migrationshintergrund [1–3]. Trotz dieser seit Jahrzehnten bestehenden Thematik existieren weiterhin keine flächendeckenden Rahmenbedingungen, um eine optimale Versorgung von Patient*innen mit Migrationshintergrund sicherzustellen. Dabei scheinen die Schaffung und Förderung von interkultureller Kompetenz des medizinischen Personals eine zentrale Bedeutung zu haben.

Ziel dieses Artikels ist es, anhand klinischer Erfahrungen eine Diskussionsgrundlage für eine stärkere Beachtung und Förderung einer nachhaltigen Auseinandersetzung mit dem Thema „interkulturelle Kompetenz“ im Gesundheitsbereich zu liefern und mögliche Handlungsfelder zu definieren. Ein interkulturelles und interprofessionelles Fortbildungsprogramm (u. a. Curriculum mit Einsatz von Simulationspatient*innen) wird vorgestellt.

## Was versteht man unter interkultureller Kompetenz?

Der Begriff „interkulturelle Kompetenz“ wird in der Literatur uneinheitlich verwendet. Im Rahmen eines internationalen Kulturdialogs der Bertelsmann Stiftung [4] wurde folgende Definition formuliert: „Interkulturelle Kompetenz ist die Fähigkeit, in interkulturellen Situationen effektiv und angemessen zu agieren; sie wird durch bestimmte Einstellungen, emotionale Aspekte, (inter-)kulturelles Wissen, spezielle Fähigkeiten und Fertigkeiten sowie allgemeine Reflexionskompetenz befördert.“ An dieser Stelle ist wichtig zu betonen, dass es sich beim Erwerb von interkultureller und Diversitätskompetenz um einen dynamischen Lernprozess handelt, der verschiedene Dimensionen umfasst [4]:

1. förderliche Einstellung,
2. umfassendes kulturelles Wissen und Fähigkeit, über interkulturelle Themen zu reflektieren,
3. die Fähigkeit, konstruktiv zu interagieren.

Um eine interkulturelle Kompetenz zu entwickeln, sind eine positive, offene Einstellung (Haltung) gegenüber interkulturellen Situationen und ein differenziertes und personalisiertes Verständnis von Kultur sehr wichtig. Der Begriff „Kultur“ in diesem Zusammenhang sollte die Inklusion aller Mitglieder einer Gesellschaft beinhalten [5] und sich mit menschlichen Interaktionen befassen, die einen Austausch „auf Augenhöhe“ ermöglichen.

Der von M. Tervalon und J. Murray-Garcia eingeführte Begriff „cultural humility“ [6], beschreibt den lebenslangen Prozess von Lernen und Selbstreflexion, patientenorientierte und an der individuellen Situation abgestimmte Gesprächsführung und Erkennen von Ungleichheiten in der Arzt-Patient-Beziehung. Ein weiterer wichtiger Aspekt sind die Mitwirkungsmöglichkeiten von sozialen Gemeinden und Patientenorganisationen im Sinne der notwendigen Partizipation. Das Recht auf Teilhabe bei Entscheidungen ist grundsätzlich notwendig, um dem Anspruch einer partizipatorischen Entscheidungsfindung für alle medizinischen Maßnahmen zu ermöglichen (Empowerment).

Hierbei ist wichtig die eigene kulturelle Prägung zu erkennen, kulturelle Aspekte selbst zu reflektieren, Gewohnheiten zu verändern sowie Vorurteile und Stereotypen einzustellen. Eine systematische Supervision bzw. ein Coaching könnte diesen notwendigen Prozess positiv unterstützen.

Die interkulturelle Kompetenz spielt zudem eine wesentliche Rolle bei der Bewältigung von medizinethischen Konflikten und kritischen medizinischen Situationen, wie sie bei der Übermittlung von schlechten Nachrichten regelhaft auftreten.

Eine gelungene Kommunikation ist die Grundlage eines kultursensitiven Agierens und beinhaltet nicht nur das Überwinden von Sprachbarrieren, sondern auch die Wahrnehmung und Wertschätzung der kulturellen Vielfalt, Offenheit und die vorurteilsfreie Begegnung mit Personen aus anderen ethnischen und kulturellen Gruppen. In diesem Zusammenhang hat Arthur Kleinman einen Fragenkatalog erstellt, um die Perspektive, die Erwartungen und laienätiologische Erklärungsmodelle der Patient*innen stärker zu berücksichtigen [7]. Um interkulturelle, interprofessionelle und diversitätssensible Kompetenzen zu fördern, sollte das Thema sowohl in der medizinischen Aus- und Weiterbildung als auch in der medizinischen Praxis kontinuierlich gelehrt und erlernt werden. Hier gibt es einen erheblichen Fort- und Weiterbildungsbedarf.

An der Charité Berlin wurde 2016 das IPIKA-Projekt als ein interkulturelles und interprofessionelles Fortbildungsprogramm entwickelt [8]. IPIKA^1^ steht für „Interprofessionelles und Interkulturelles Arbeiten in Medizin, Pflege und Sozialdienst“. Das Projekt wurde in Kooperation mit der Klinik für Gynäkologie, dem Comprehensive Cancer Center der Charité und der Alice Salomon Hochschule (ASH) gestartet und durch die Robert Bosch Stiftung gefördert. In einer weiteren Förderphase (2018–2019) konnte das IPIKA-Curriculum im Bildungsprogramm der Charité Fortbildungsakademie etabliert und ein IPIKA-Zertifikatskurs in das Weiterbildungsprogramm der ASH aufgenommen werden. Wie im Artikel unserer Arbeitsgruppe für die Zeitschrift *FORUM* beschrieben [8], handelt es sich dabei um eine transprofessionelle und interdisziplinäre Fortbildung, die auf verschiedene Berufsgruppen aus Pflege, Medizin, Psychoonkologie, Sozialdienst und Hebammen ausgerichtet ist. Sie vermittelt berufsübergreifend praxisnahe interkulturelle Kompetenzen und Kommunikationsfähigkeit und wurde inzwischen als Regelfortbildung der Mitarbeiter*innen eingeführt.

Das Fortbildungsprogramm umfasst 6 2-tägige Module und beinhaltet folgende Themen [8].

- Zusammenhänge zwischen Migration, Flucht und (seelischer) Gesundheit, die in Diagnostik, Anamnese, Pflege und Therapie relevant sein können;
- soziokulturell beeinflusste subjektive Vorstellungen zu Krankheit, Behandlung und Bewältigungsformen, die oft den Behandlungs- und Pflegeprozess beeinflussen;
- Umgang mit Sprachbarrieren, Einsatz von professionellen Sprachmittler*innen, Arbeit mit Video- und Telefondolmetscher*innen, Nutzung von Bildtafeln, Kommunikations- und Übersetzungs-Apps;
- Auseinandersetzung mit (eigenen) Stereotypen, Vorurteilen und Fremdbildern, Handlungsmöglichkeiten gegen Diskriminierung und Rassismus im Klinikalltag;
- Umgang mit ethischen Fragen in interkulturellen Kontexten, beispielweise bei Patientenaufklärung, Entscheidungen am Lebensende, Einflussnahme von Angehörigen;
- Deeskalation und Konfliktbewältigung in interkulturellen Überschneidungssituationen, Erweiterung der eigenen Handlungsoptionen;
- interprofessionelle Zusammenarbeit und Vernetzung.

Verschiedene Studien zum Thema Inanspruchnahme von Früherkennungsmaßnahmen bei Menschen mit Migrationshintergrund belegen, dass Migrant*innen mit verschiedenen Barrieren konfrontiert werden. Ergebnisse einer Studie unserer Arbeitsgruppe an 606 Patientinnen mit gynäkologischen Malignomen zeigen, dass Patient*innen mit Migrationshintergrund u. a. signifikant seltener die Teilnahme an klinischen Studien zu Krebstherapien angeboten wird [9]. Eine Befragung von 552 Patient*innen mit Kolonkarzinom der Arbeitsgruppe von Leonhardt et al. zeigte zudem, dass Patient*innen mit Migrationshintergrund seltener in Tumornachsorgeprogramme eingebunden waren und weniger zufrieden mit ihrer Beteiligung an der Entscheidungsfindung waren [10]. Ursachen dieser Zugangsbarriere zu klinischen Studien können nicht nur die Sprachbarriere, sondern auch Kommunikationsdefizite in der Arzt-Patienten-Kommunikation sein und des Weiteren fehlende Strukturen, wie z. B. die Möglichkeit, routinemäßig Dolmetscher*innen beim Aufklärungsgespräch einzusetzen, oder fehlende spezifische Informations- und Aufklärungsmaterialien in der jeweiligen Sprache.

Auch bei Entscheidungen am Lebensende und in der palliativen Phase zeigen sich Unterschiede zwischen Nichtmigrant*innen und Migrant*innen. In einer aktuellen Befragung von 736 Frauen mit gynäkologischen Krebserkrankungen von Inci et al. zum Thema Patientenverfügung und Vorsorgevollmacht zeigte sich, dass Migrantinnen signifikant weniger über das Thema Patientenverfügung informiert wurden und sich mehr Information in der eigenen Sprache wünschten (Daten noch nicht publiziert). Wie von Ilkilic et al. [11] formuliert, sollte eine kultursensible und kultursensitive Patientenverfügung in der Lage sein, kulturspezifische Wertvorstellungen und Wünsche am Lebensende verständlich, deutlich und in anwendbarer Form auszudrücken.

Viele Konflikte und Probleme ergeben sich aus einer unzureichenden Kommunikation und sind nicht kulturspezifisch erklärbar. Daher sollte der Schwerpunkt auch in den verschiedenen medizinischen Fortbildungs- und Weiterbildungsprogrammen auf die gesamten Aspekte der Kommunikation gerichtet sein. In Tab. 1 sind die Grundprinzipien einer interkulturellen Arzt-Patienten-Kommunikation zusammengefasst.

**Table 1:** 

1	Lassen Sie sich Zeit, Ihre eigenen Reaktionen und die der Patient*innen wahrzunehmen, und versuchen Sie, eigenen stereotypen Denk- und Handlungsweisen vorzubeugen, diese zu identifizieren und ihnen entgegenzusteuern. Nehmen Sie jemanden aus Ihrem Team mit, um den Blick von außen zu nutzen
2	Bei Anwesenheit von Angehörigen bzw. Dolmetscher*innen sollte die Sitzordnung so gewählt werden, dass Ärztin/Arzt und Patient*innen direkten Augenkontakt haben. Achten Sie dabei besonders auf die nonverbale Kommunikation
3	Vergewissern Sie sich vor dem Gespräch über die Kompetenz der Dolmetscher*innen und deren Beziehung zu den Patient*innen. Informieren Sie die Dolmetscher*innen in einem kurzen Vorgespräch über das Ziel des Gesprächs und die verschiedenen Rollen
4	Bei wiederkehrenden Gesprächen bitten Sie darum, dass möglichst dieselbe Dolmetscher*in bzw. dieselben Angehörigen in die Gespräche eingebunden sind
5	Erklären Sie zu Beginn das Ziel des Gesprächs und loten Sie die Erwartungen der Patient*innen aus. Kommunizieren Sie Ihre Erwartungen an die Patient*innen und die anderen anwesenden Personen und geben Sie auch den Erwartungen der Patient*innen und Angehörigen Zeit und Raum
6	Setzen Sie professionelle Sprachmittler*innen ein, die für die Sprachmittlung im Gesundheitswesen qualifiziert sind
7	Formulieren Sie Ihre Informationen und Botschaften klar und nutzen Sie eher direkte als indirekte Kommunikation. Geben Sie den Sprachmittler*innen für die Übermittlung und den Patient*innen für etwaige Gegenfragen genügend Zeit
8	Konzentrieren Sie sich bewusst und gezielt auf vertrauensstiftende Maßnahmen und den Auf- und Ausbau der Arzt-Patienten-Beziehung. Laden Sie beispielsweise die Patient*innen wiederholt dazu ein, Fragen zu stellen, und fragen Sie aktiv nach, was bei den Patient*innen inhaltlich angekommen ist
9	Überfrachten Sie das Gespräch nicht mit zu vielen Informationen und Aufgaben

Vorurteile, die bewusst oder unbewusst vorliegen können, erschweren grundsätzlich die Kommunikation. Sie sollten in der Eigenwahrnehmung sichtbar gemacht und artikuliert werden. Dabei sind alle Facetten des „Andersseins“ wie Geschlecht, Alter, Berufsgruppe, Krankheitsgruppe und Religion zu berücksichtigen. Diversität ist ein Kernthema für die Entwicklung einer adäquaten Gesundheitsversorgung, die eine Teilhabe und Chancengleichheit für alle Menschen zum Ziel hat.

Die bisherigen Erfahrungen mit der Implementierung von interkulturellen und diversitätssensiblen Kompetenzen stammen häufig aus lokalen Projekten und Initiativen. Ziel sollte es aber sein, ein strukturiertes und nachhaltiges interdisziplinäres, interprofessionelles und sektorenübergreifendes Netzwerk sowohl im klinischen als auch im wissenschaftlichen Bereich zu etablieren. In einer Arbeit von Stadler et al. [13] wurde ein „*Diversitäts-MindestIndikatorenSatz (DiMIS)*“ definiert, mit dessen Hilfe verschiedene Diversitätsdomänen (Geschlecht, Alter, Bildungsstatus, Sozialstatus, sexuelle Orientierung, Herkunft, Ethnizität, Religion, körperliche und mentale Gesundheit, Behinderungen) und Diskriminierungserfahrungen in Studien erfasst werden können, um eine prospektive und systematische wissenschaftliche Bearbeitung zu ermöglichen.

Ein vor Kurzem gestartetes Projekt an der Charité Berlin, das von der Stiftung „Mercator“ gefördert wird, mit dem Namen „Empowerment für Diversität – Kompetenzen und Strukturen für Diversitätsgerechtigkeit und Chancengleichheit in der Gesundheitsversorgung“^2^, hat dabei als wesentliches Ziel die Etablierung eines nachhaltigen, interprofessionellen und interdisziplinären bundesweiten Netzwerks von Expert*innen, Institutionen und politischen Entscheidungsträger*innen, das sich dauerhaft und über den Projektzeitraum hinausgehend für Chancengleichheit und Diversitätsgerechtigkeit im deutschen Gesundheitssystem einsetzt. Dieses Aktionsbündnis soll die bestehenden Kompetenzen zu einer nachhaltigen Allianz bündeln, um das verfügbare Wissen in die klinische Versorgung zu überführen. Es soll diversitätsgerechte Strukturen und Kompetenzen im deutschen Gesundheitssystem flächendeckend etablieren und fördern sowie bestehenden Diskriminierungsrisiken und Zugangsbarrieren entgegenwirken, von denen insbesondere Menschen mit Migrations- und Fluchtgeschichte sowie Black, Indigenous und People of Color (BIPoC) betroffen sind.

## Fazit

Interkulturelle und Diversitätskompetenz sollten zu den Kernkompetenzen aller Berufsgruppen im Gesundheitswesen zählen. Dies ist eine der Grundvoraussetzungen für partizipative Entscheidungen und eine vollwertige Gesundheitsversorgung. Die berufsgruppenübergreifende Verankerung von systematischen Fort- und Weiterbildungsprogrammen (z. B. das IPIKA-Konzept) mit dem Ziel der Entwicklung und Förderung von interkultureller Kompetenz kann hierbei eine zentrale Rolle für eine grundsätzliche Veränderung im Gesundheitswesen spielen.

## Footnotes

1 https://interkulturelle-kompetenz.charite.de/projekte/ipika/; Zugriff: 23.08.2023.

2 https://diversity.charite.de/diversity_projekte/empowerment_fuer_diversitaet/; Zugriff: 23.08.2023.
